# The immunophilin repertoire of *Plasmodiophora brassicae* and functional analysis of *PbCYP3* cyclophilin

**DOI:** 10.1007/s00438-017-1395-0

**Published:** 2017-11-11

**Authors:** Khushwant Singh, Georgios Tzelepis, Miloslav Zouhar, Pavel Ryšánek, Christina Dixelius

**Affiliations:** 1 Department of Plant Biology, Uppsala BioCenter, Linnean Centre for Plant Biology, Swedish University of Agricultural Sciences, P.O Box 7080, 75007 Uppsala, Sweden; 2 Department of Plant Protection, Faculty of Agrobiology, Food and Natural Resources, Czech University of Life Sciences Prague, Prague, Czech Republic; 3 Division of Crop Protection and Plant Health, Crop Research Institute, Drnovska 507, 16106 Prague, Czech Republic

**Keywords:** Cyclophilin, Immunophilin, *Plasmodiophora brassicae*, Rhizaria

## Abstract

*Plasmodiophora brassicae* is a soil-borne pathogen that belongs to Rhizaria, an almost unexplored eukaryotic organism group. This pathogen requires a living host for growth and multiplication, which makes molecular analysis further complicated. To broaden our understanding of a plasmodiophorid such as *P. brassicae*, we here chose to study immunophilins, a group of proteins known to have various cellular functions, including involvement in plant defense and pathogen virulence. Searches in the *P. brassicae* genome resulted in 20 putative immunophilins comprising of 11 cyclophilins (CYPs), 7 FK506-binding proteins (FKBPs) and 2 parvulin-like proteins. RNAseq data showed that immunophilins were differentially regulated in enriched life stages such as germinating spores, maturing spores, and plasmodia, and infected *Brassica* hosts (*B. rapa, B. napus* and *B. oleracea*). *PbCYP3* was highly induced in all studied life stages and during infection of all three *Brassica* hosts, and hence was selected for further analysis. *PbCYP3* was heterologously expressed in *Magnaporthe oryzae* gene-inactivated Δ*Cyp1* strain. The new strain Δ*Cyp1*+ overexpressing *PbCYP3* showed increased virulence on rice compared to the Δ*Cyp1* strain. These results suggest that the predicted immunophilins and particularly *PbCYP3* are activated during plant infection. *M. oryzae* is a well-studied fungal pathogen and could be a valuable tool for future functional studies of *P. brassicae* genes, particularly elucidating their role during various infection phases.

**Electronic supplementary material:**

The online version of this article (10.1007/s00438-017-1395-0) contains supplementary material, which is available to authorized users.

## Introduction

The plant immune system recognizes microbial pathogens in various ways to prevent infection. Pathogens try on the other hand to evade recognition and defense responses in plants. Knowledge on these events has rapidly increased over the last years (Boutrot and Zipfel 2017). However, pathogens with obligate biotrophic lifestyle are lagging behind in this context, since extraction of high-quality nucleic acids and any kind of gene editing are difficult and in some cases almost impossible. *Plasmodiophora brassicae* is a good example of this type of category. This organism is an obligate biotrophic plasmodiophorid that belongs to the Phytomyxea class within the eukaryote supergroup Rhizaria, taxonomically distinct from other plant pathogens, such as fungi or oomycetes (Neuhauser et al. 2011; Sierra et al. 2016). Rhizaria is one of the least studied groups of eukaryotes (Sibbald and Archibald 2017). Besides *P. brassicae*, few other genomes are available in Rhizaria; all for diverse species such as the chlorarachniophyte alga *Bigelowiella natans*, the foraminifera *Reticulomyxa filosa*, the transcriptome of the potato powdery scab pathogen *Spongospora subterranea* and few transcriptome datasets on marine species (Curtis et al. 2012; Glöckner et al. 2014; Keeling et al. 2014; Schwelm et al. 2015; Krabberød et al. 2017). The *P. brassicae* genome is relatively small (25.5 Mb) compared to the free-living *B. natans* (~ 100 Mb) and *R. filosa* (~ 320 Mb). Plasmodiophorids infect a wide range of host organisms (Neuhauser et al. 2014). The Brassicaceae plant family is the preference of *P. brassicae*, the clubroot disease agent. This disease is increasing in importance, causing a 10–15% yield reduction on a global scale (Dixon 2009). Due to the hidden lifestyle in the soil and its requirement of a living host plant root for growth and multiplication, many aspects on this plant pathogen remain to be elucidated. This also calls for new tools for functional gene assessments.

Immunophilins are ubiquitous proteins with properties that allow them to regulate protein structure, activity and stability (Wang and Heitman 2005; Hanes 2015). They operate either by peptidyl-propyl isomerization of selected targets, as chaperons or by binding of small ligands. The immunophilins comprise of three structurally unrelated subfamilies: the cyclophilins (CYPs), the FK506-binding proteins (FKBPs), and the parvulin-like proteins (Hanes 2015). Besides modulating the formation of cis–trans isomers of proline (Galat 2003), immunophilins have two important amino acid properties: (a) prolyl-isomerase activity, which catalyzes the rotation of the X-Pro peptide bonds from the cis to trans configuration, a rate-limiting step in protein folding (Wang and Heitman 2005), and (b) affinity to bind to immunosuppressive drugs. The stramenopile human parasite *Blastocystis* sp. lives under anaerobic conditions partly like *P. brassicae* (Gravot et al. 2016) and secretes a range of immunophilins with potential effector functions, which could lead to cell death (apoptosis) in the host tissue (Denoeud et al. 2011). Another animal parasite, the protozoan *Toxoplasma gondii* uses a cyclophilin (TgCYP18) to manipulate host cell responses (Ibrahim et al. 2009). Among plant pathogens, some cyclophilins are able to inhibit RNA replication of plant viruses (Lin et al. 2012; Kovalev and Nagy 2013), while some effector proteins interact with plant cyclophilins, and thereby induce plant defense responses (Domingues et al. 2010, 2012; Kong et al. 2015). In the rice blast fungus *Magnaporthe oryzae*, the cyclophilin A homolog MgCYP1 acts as a virulence determinant. When this gene was inactivated it led to reduced virulence, and the *Cyp1* mutant strain was malfunctioned regarding formation of penetration peg and appressorium turgor generation (Viaud et al. 2002). MgCYP1 is also the cellular target for the drug cyclosporin A in *M. oryzae*, functioning as an inhibitor of appressorium development and hyphal growth in a CYP1-dependent manner, suggesting a role for the calcineurin regulation of appressorium development (Viaud et al. 2002).

This work aimed to monitor the presence of immunophilin encoding genes in the genome of *P. brassicae*, and to analyze potential candidates for function, here using the molecular amenable plant pathogen *M. oryzae* as heterologous test system. All with the overall aim of enhancing our understanding of this root gall (club)-inciting plant pathogen.

## Materials and methods

### Prediction of immunophilins, domain analysis and subcellular localization

To identify putative members of immunophilins in *P. brassicae*, the Hidden Markov Model profiles, unique to cyclophilin (PF00160), FKBP (PF00254) and parvulins (PF00639) from the Pfam database version 28.0 (Finn et al. 2016), were retrieved and searched against the annotated genome of *P. brassicae* (Schwelm et al. 2015). Protein candidates were identified as described previously (Singh et al. 2014; Tripathi et al. 2015). Significant hits (*e* value: 1.0E–0) with positive scores were selected for further classification. In analogy with earlier denominations, the proteins identified were named with the prefix Pb (*P. brassicae*), followed by CYP (cyclophilin), FKB (FK506-binding proteins), and PAR (parvulin-like proteins), according to the catalytic domain present, followed by numbers in increasing order based on the highest Hidden Markov Model scores. Protein domain structures were confirmed with the SMART software (Letunic et al. 2015).

Subcellular localization of the putative immunophilin proteins was predicted using PSORT (Nakai and Horton 1999). Signal peptide cleavage sites and mitochondrial-targeted peptides were predicted using SignalP 4.1 (Petersen et al. 2011) and the TargetP 1.1 server, respectively (Emanuelsson et al. 2000). Nuclear localization signals (NLS) were predicted using NLS mapper (Kosugi et al. 2009).

### Phylogenetic analysis

Phylogenetic analysis was conducted on PbCYP3 homologs, derived from different phytopathogens and the host *Arabidopsis thaliana*, based on catalytic domain sequences and was carried out using the maximum likelihood method implemented in the MEGA v.7 software (Kumar et al. 2016), using the JTT substitution model (Jones et al. 1992). Bootstrap analysis was performed on 1000 replicates. The CLUSTAL W algorithm was used for alignments (Thompson et al. 1994) (Supplementary dataset 1).

### Transcriptome analysis

The transcriptome of *P. brassicae* genes, coding for putative immunophilins, was analyzed exploiting data from various life-stage-specific forms, such as germinating spores, maturing spores and plasmodia of *P. brassicae* and in clubroot-infected *Brassica* hosts (*B. rapa, B. napus* and *B. oleracea*), as described by Schwelm et al. (2015). Fragments per kilobase of transcript per million mapped reads (FPKM) were calculated (Trapnell et al. 2010). Furthermore, the immunophilin gene expression levels were visualized in heat maps as log10-transformed FPKM values and were normalized by calculating the *Z*-score for each gene across all transcriptome libraries. Heat maps were drawn using the gplot package of R software (R Core Team 2014), as described previously by Singh et al. (2014).

### Construction of overexpression vector and *Magnaporthe oryzae* transformation

The *P. brassicae* single spore isolates e3, was used for spore isolation and purification (Fähling et al. 2004). DNA was extracted from purified *P. brassicae* resting spores using a NucleoSpin® Plant II Mini kit (Macherey–Nagel) following manufacturer’s instructions. *PbCYP3* was PCR amplified from genomic DNA using high fidelity Phusion Taq polymerase (Thermo Scientific) and *Bam*HI*PbCyp3* and *Kpn*I*PbCyp3* primers (Table 1). The pCB1532 vector, conferring resistance to chlorimuron ethyl (Yang and Naqvi 2014), was used as a destination vector to construct the pCB1532-PbCYP3+ overexpression vector. The orientation and integrity of the insertion were confirmed by DNA sequencing (Macrogen Inc.).

**Table 1:** List of primers used in the current study

Primer name	5′–3′
BamHIPbCyp3	ATATGGATCCATGGCGAACCCGAAGGTCT
KpnIPbCyp3	ATATCCATGGTCAGCACTCGCCGCACTTC
OsElfF	TTGTGCTGGATGAAGCTGATG
OsElfR	GGAAGGAGCTGGAAGATATCATAGA
MgActF	ATGTGCAAGGCCGGTTTCGC
MgActR	TACGAGTCCTTCTGGCCCAT
PbCYP3C_RT-F	ATTTCACGAACCACAACGGCACTG
PbCYP3C_RT-R	TGGACACGGTGCACACGAAGAAC

Construction of *M. oryzae* Δ*Cyp1*+ overexpression strain (containing the *P. brassicae PbCYP3* gene) was carried out using a protoplast-mediated protocol. Briefly, *M. oryzae* mycelia from the Δ*Cyp1* strain (Viaud et al. 2002), with the Guy11 genomic background, were incubated in OM buffer (1.2 M MgSO_4_, 10 mM Na-PO_4_ pH 5.8) containing lytic enzymes (Novozymes). Protoplasts were mixed with the pCB1532-PbCYP3+ overexpression vector in STC buffer (1.2 M sorbitol, 10 mM Tris–HCl pH 7.5, 10 mM CaCl_2_) and incubated at room temperature for 25 min. PTC buffer (60% PEG 4000, 10 mM Tris–HCl pH 7.5, 10 mM CaCl_2_) was then added followed by incubation at room temperature for 20 min. Finally, protoplasts were added to molten BDC medium (yeast nitrogen base without amino acids and ammonium sulfate, 1.7 g/L ammonium nitrate, 2 g/L, asparagine, 1 g/L, glucose, 10 g/L, pH 6, 1.5% agar). The plates were incubated for at least 16 h at 24 °C and overlaid with approximately 15 ml BDC medium without sucrose, supplemented with 1.5% agar and 150 μg mL^−1^ chlorimuron ethyl (Sigma Aldrich). Fungal colonies resistant to chlorimuron ethyl were verified by PCR that contain the *PbCYP3* gene, and expression levels of this gene confirmed using RT-qPCR as described below.

### Quantitative real-time PCR

Total RNA was extracted from 1-week-old cultures of wild-type (Guy 11), Δ*Cyp1* and Δ*Cyp1+ M. oryzae* strains using the RNeasy Plant Mini Kit (Qiagen) according to manufacturer’s instructions and concentrations were determined using NanoDrop (Thermo Scientific). For cDNA synthesis, 1 µg total RNA was reversed transcribed in a total volume of 20 µl using the iScript cDNA Synthesis Kit (Bio-Rad). Transcript levels were quantified by quantitative reverse transcriptase PCR (RT-qPCR) using the iQ5 qPCR System (Bio-Rad) as described previously (Tzelepis et al. 2012). Relative expression values of *PbCYP3* gene were calculated using the 2^−ΔΔCT^ method (Livak and Schmittgen 2001). The *M. oryzae* actin gene (Che Omar et al. 2016) was used to normalize the data using the MgActF/R primers listed in Table 1.

### Phenotypic analysis and quantification of *Magnaporthe oryzae* biomass in infected plants

*Magnaporthe oryzae* Guy11 (WT), Δ*Cyp1*+ and Δ*Cyp1* strains were grown in triplicates on oatmeal agar plates and kept for 1 week at 25 °C in darkness. Rice plants of the cultivar CO-39 (*Oryza sativa*) were grown under controlled conditions at 28 °C in cycles of 14 h light and 10 h dark. 4-week-old plants were inoculated with 2 mm mycelia plugs, derived from 2-week old *M. oryzae* cultures of wild-type, Δ*Cyp1*+ and Δ*Cyp1*, while mock inoculation was conducted with only agar plugs as previously described (Dong et al. 2015). Fungal colonization on leaves were monitored after 1 week and DNA was extracted using a CTAB method (Möller et al. 1992) and quantified using the *M. oryzae* actin (*act*) gene, normalized with the elongation factor gene (*elf-1*) from *Oryza sativa* (Ma et al. 2011) and qPCR techniques as described above. At least five biological replicates were used. For the statistical analyses, the ANOVA (one way) was conducted using a general linear model implemented in SPSS 20 (IBM). Pairwise comparisons were performed using the Tukey’s method at the 95% significance level (Tukey 1949).

## Results

### Sequence and structure characteristics of *Plasmodiophora brassicae* immunophilins

**Fig. 1:**
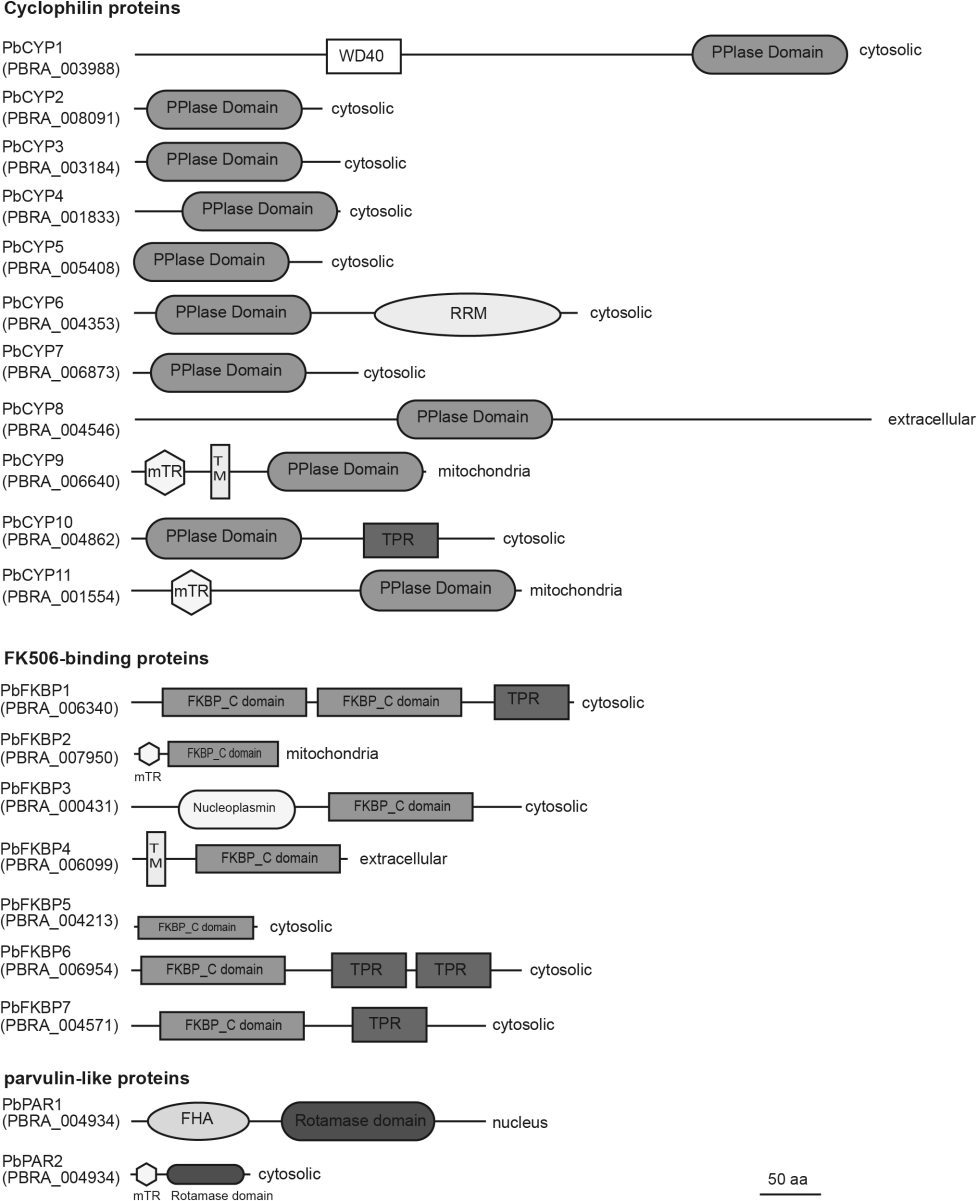
Domain architecture of predicted immunophilins in *Plasmodiophora brassicae* genome. Amino acid sequences were analyzed for the presence of conserved domains using the SMART software. *WD40* tryptophan-aspartic acid repeats, *PPIase* peptidyl-prolyl isomerase, *RRM* RNA recognition motif, *FKBP* FK506-binding protein, *TPR* tetracopeptide repeat, *FHA* forkhead-associated domain, *rotamase domain* parvulin-like rotamase domain, *mTP* mitochondrial target peptide, *TM* transmembrane domain. Predicted cell localization is indicated. The bar marker indicates a length of 50 amino acids and refers to total protein length

Our analysis revealed that the *P. brassicae* genome contained 20 genes encoding putative immunophilins (Fig. 1). Eleven belong to the cyclophilins, seven to FKBP and two to the parvulin subfamilies. Seven proteins carried a single catalytic domain, while a transmembrane domain was predicted for two. Only the PbCYP1 protein harbored a WD-40 domain, while a nucleoplasmin domain was detected in the PbFKBP3 sequence. Four proteins harbored tetracopeptide repeats, while PbPAR1 and PbCYP6 each contained a forkhead-associated or a RNA recognition motif. Based on localization signals, following distribution was predicted; PbCYP8 and PbFKBP4 were extracellular, PbPAR1 was localized to the nucleus, while PbCYP9, PbCYP11 and PbFKBP2 were localized to the mitochondria. The remaining 14 proteins predicted to be localized in the cytosol (Fig. 1).

### Expression patterns of immunophilin genes in various *P. brassicae* stages and clubroot-infected plants

**Fig. 2:**
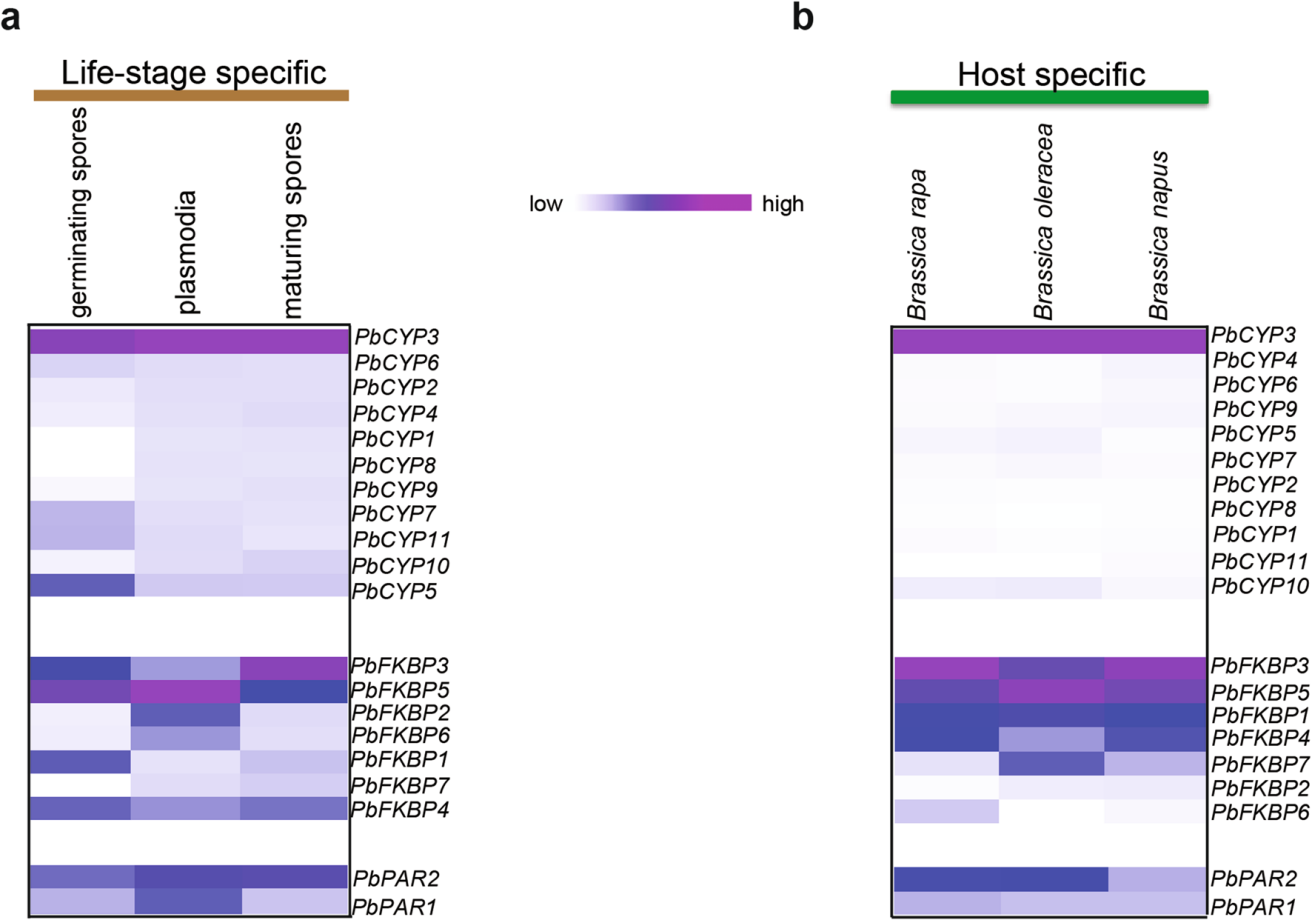
Expression patterns of the predicted immunophilins (IMMs) from *P. brassicae*. The heat maps show the RNAseq-based expression profile of IMMs during: **a** different *Plasmodiophora brassicae* life stages; germinating spores, plasmodia and maturing spores, and **b** different clubroot-infected *Brassica* hosts; *B. rapa, B. napus* and *B. oleracea*. The heat maps were drawn using the gplot package of R statistical software

Re-analyzed RNAseq data from Schwelm et al. (2015) generated differential expression patterns for genes, encoding for putative immunophilins, in various enriched life stages, such as germinating and maturing spores, plasmodia and clubroot-infected *Brassica* hosts (*B. rapa, B. napus* and *B. oleracea*). Notably, *PbCYP3* was highly induced in all studied life stages (Fig. 2a), while *PbCYP5, PbCYP7* and *PbCYP11* showed elevated transcript levels in germinating spores. The other *PbCYP* genes had low transcript levels. Only the *PbCYP3* gene displayed high induction during infection of the three *Brassica* hosts, while others showed low expression levels (Fig. 2b). In the FKBP subfamily, *PbFKBP3* and *PbFKBP5* were highly activated during different life stages. *PbFKBP2* and *PbFKBP6* were upregulated in plasmodia, and *PbFKBP1* during the germinating spore stage (Fig. 2a). Notably, *PbFKBP3* and *PbFKBP5* were highly induced in all host species, whereas *PbFKBP7* was highly induced only in *B. oleracea* (Fig. 2b). In the case of the PAR subfamily, *PbPAR2* was constitutively expressed during all stages compared to *PbPAR1* (Fig. 2a). High transcript level of *PbPAR2* was also observed in *B. rapa* and *B. oleracea* compared to *B. napus*, while *PbPAR1* was expressed at low levels in all *Brassica* hosts.

### Sequence, phenotypic and pathogenicity analyses of *PbCYP3*

**Fig. 3:**
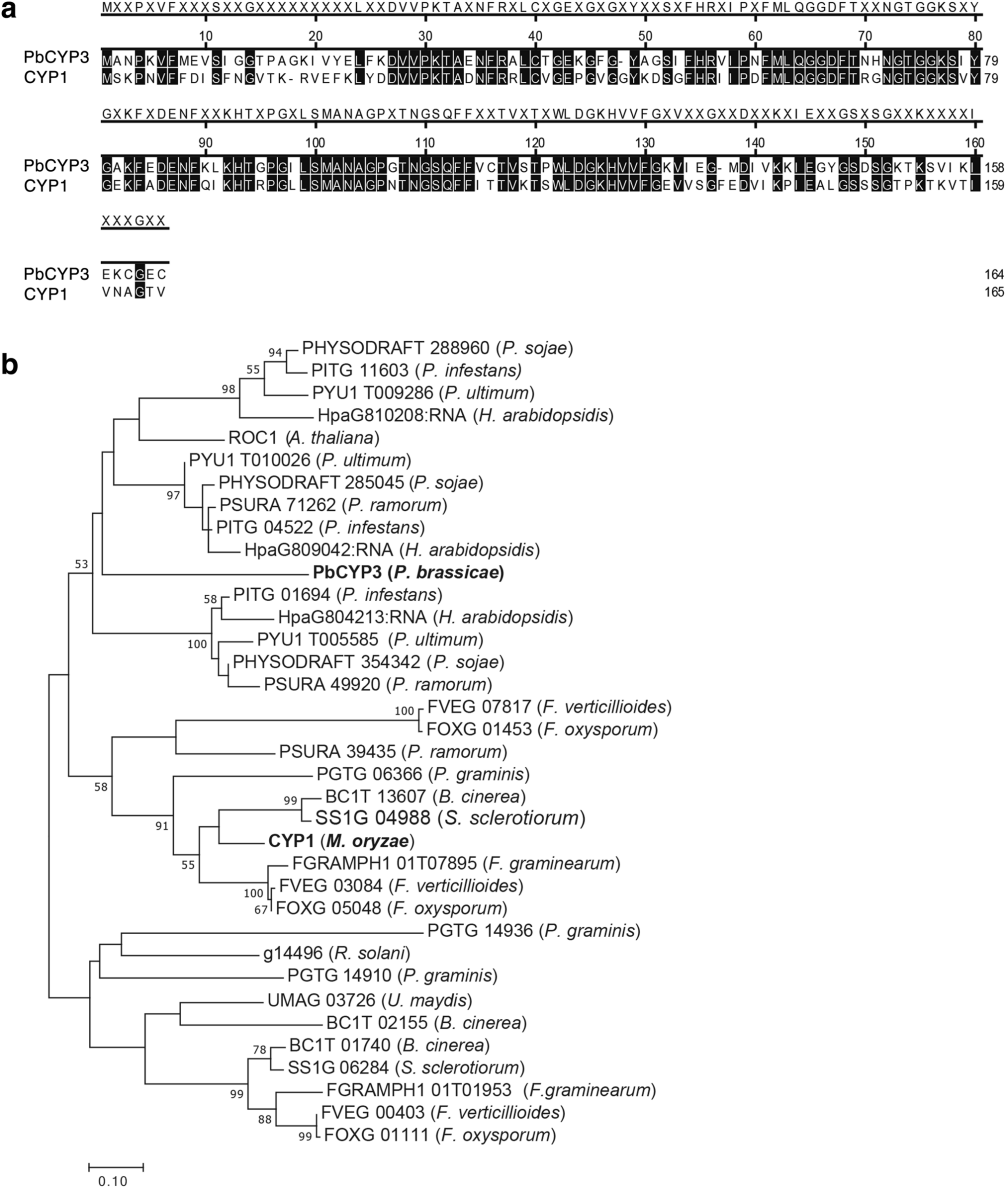
Comparison of *Plasmodiophora brassicae* PbCYP3 and *Magnaporthe oryzae* CYP1. **a** Amino acid alignment between PbCYP3 and CYP1. Sequence similarities are depicted with black shade. Alignments were conducted using the CLUSTALW algorithm implemented in MegAlign software (DNASTAR, Madison). **b** Phylogeny of homologs to the PbCYP3 cyclophilin. Analysis was conducted using maximum likelihood with the JTT substitution model, based on Clustal W alignment using all amino acid sites. The bar marker indicates the numbers of amino acid substitutions. Protein identifiers include protein name (if available) or protein ID accession numbers from TAIR and FungalDB databases. *Plasmodiophora brassicae* and *Magnaporthe oryzae* cyclophilins are indicated in bold. Bootstrapping was with 1000 replicates and values ≥ 50 are shown

In our sequence comparison, the *P. brassicae* PbCYP3 cyclophilin showed 60% similarity to the *M. oryzae* CYP1 (Fig. 3a). In phylogenetic analysis of PbCYP3 homologs present in plant pathogenic fungi and oomycetes, PbCYP3 grouped together with homologs from oomycetes (Fig. 3b), while *M. oryzae* CYP1 clustered in a separate group together with cyclophilins derived from filamentous ascomycetes.

**Fig. 4:**
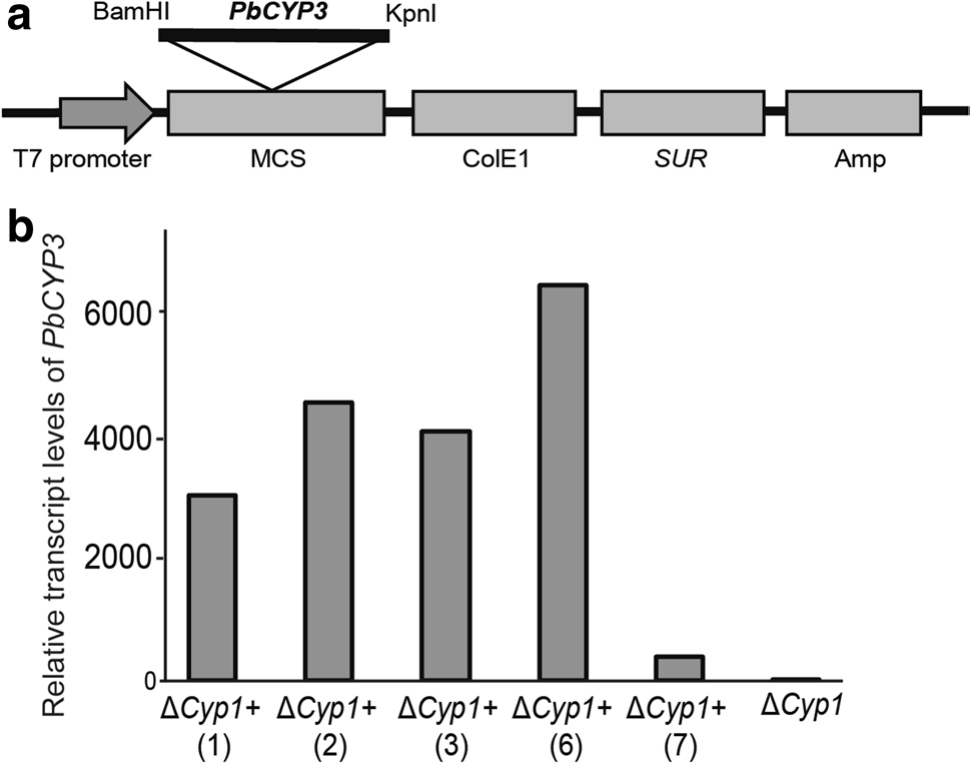
Construction and validation of *Magnaporthe oryzae* Δ*Cyp1*+ strain. **a** Map of pCB1532:PbCyp3+ vector used for *M. oryzae* transformation. The plasmid confers resistance to chlorimuron ethyl (SUR) in *M. oryzae* and to ampicillin (Amp) in bacteria. **b** Expression profiles of the *PbCYP3* gene in five (1–7) positive Δ*Cyp1*+ transformants. Relative transcription levels in relation to actin gene (*act*) expression are calculated from Ct values and according to DDCt method

**Fig. 5:**
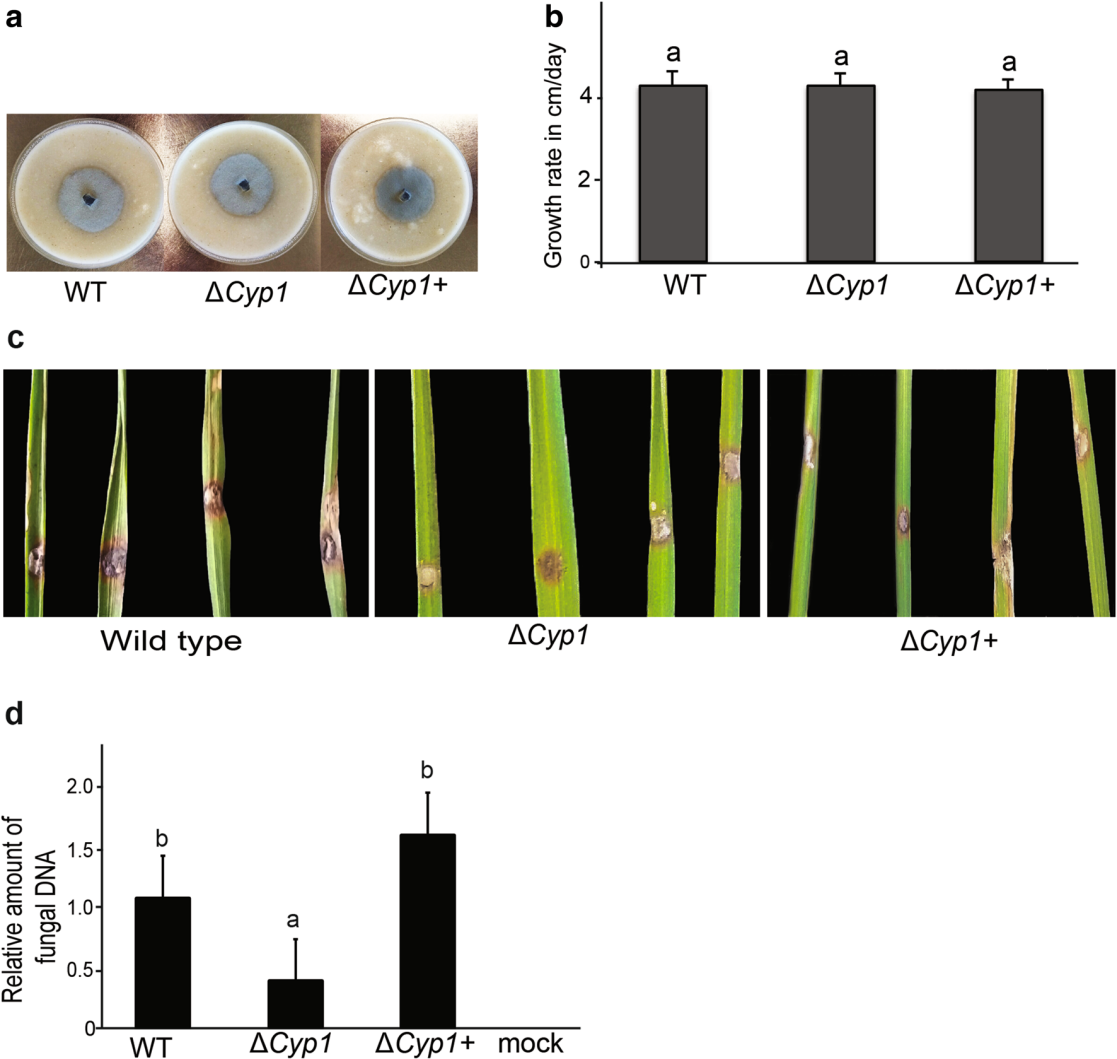
Functional analysis of *Magnaporthe oryzae* strains. **a** Colony morphology of *M. oryzae* Guy11 wild-type (WT), Δ*Cyp1* and Δ*Cyp1*+ strains grown on oatmeal agar. Cultures were maintained for 2 weeks at 25 °C in darkness. **b** Growth rate of *M. oryzae* WT, Δ*Cyp1* and Δ*Cyp1*+ strains grown on oatmeal agar. **c** Symptoms of *M. oryzae* WT, Δ*Cyp1* and Δ*Cyp1*+ strains on rice plants 7dpi. **d** Biomass quantification of *M. oryzae* WT, Δ*Cyp1* and Δ*Cyp1*+ strains upon infection of rice plants cv. CO-39. DNA was extracted from plants 7 days post infection. For quantitative PCR (qPCR), the *M. oryzae* actin (*act*) gene was used and data were normalized with the elongation factor gene (*elf-1*) from *Oryza sativa*. Letters (a, b) indicate statistically significant differences (*p* value < 0.05) according to Tukey’s HSD test. Error bars represent SD based on at least five biological replicates

Heterologous expression of the *PbCYP3* in the *M. oryzae* deletion strain Δ*Cyp1* was produced using the pCB1532:PbCyp3+ vector (Fig. 4a). Five selected chlorimuron ethyl-resistant *M. oryzae* transformants showed elevated transcript levels of the *PbCYP3* gene (Fig. 4b). The Δ*Cyp1*+ transformant (no. 6), which showed the highest transcript levels, was used for further analysis. No difference in colony morphology and mycelial growth rate was observed between *M. oryzae* Guy11 (wild-type), Δ*Cyp1*, and the Δ*Cyp1*+ strains (Fig. 5a, b). To evaluate the role of *PbCYP3* in virulence, rice plants were infected with these three strains independently. Our results showed that the wild-type strain caused more severe symptoms on rice plants compared to symptoms caused by the Δ*Cyp1* and Δ*Cyp1*+ strains (Fig. 5c). However, no significant differences between wild-type and the Δ*Cyp1*+ strain were observed in fungal biomass 7 days post infection, whereas the Δ*Cyp1* strain had significantly lower DNA levels, signifying reduced plant colonization ability (Fig. 5d).

## Discussion

The immunophilin repertoire in *P. brassicae* comprises 20 putative members, distributed on three subfamilies. This gene family size is also common in other eukaryotes such as nematodes, fungi, and oomycetes (Page et al. 1996; Pemberton 2006; Gan et al. 2009; Krucken et al. 2009; Singh et al. 2017). Commonly, plants harbor 50–60 immunophilin proteins (Vasudevan et al. 2015). *Arabidopsis thaliana*, a host of *P. brassicae* has 52 immunophilins (He et al. 2004). Much of immunophilin gene function in plants is associated to development but also to different abiotic and stress responses. The *P. brassicae* nuclear genome lacks a number of gene coding for essential metabolites (Schwelm et al. 2015), which is expected to be a feature of the poorly understood biotrophic lifestyle where the pathogen has evolved a strict dependency with its host (Kemen et al. 2011). Whether there is any overlapping function between the *CYP* genes in *P. brassicae* and those in the host is, as most gene functions in this specific plant–pathogen interaction, not known. The PbCYP3 homolog in *A. thaliana* is the cyclophilin ROC1 (AT4G38740), a gene known to function in the immune response pathways to *Pseudomonas syringae* (Aumüller et al. 2010). ROC1 also takes part in brassinosteroid signaling (Trupkin et al. 2012). Brassinosteroid synthesis and signaling in infected *A. thaliana* plants participates in clubroot formation (Schuller et al. 2014). It has been suggested that biotrophic pathogens can manipulate the host dependency to promote brassinosteroid levels favorable for infection (Belkhadir et al. 2012). Thus, it cannot be excluded that PbCYP3 is active in a similar process which also could explain the high transcript levels observed in the infected Brassica host.

Heterologous expression of hormone-encoding genes isolated from *P. brassicae* has earlier taken place using *Escherichia coli* followed by in vitro activity tests (Schuller and Ludwig-Müller et al. 2006, 2015). Here, we tested the *PbCYP3* function in *M. oryzae* system since *P. brassicae* is not amenable for such analysis. *M. oryzae* is a hemibiotrophic pathogen that causes blast disease on rice and other grass species. After plant cell penetration, the fungus first proliferates inside living host cells (biotrophic stage) followed by necrotrophic growth feeding on dead tissue (Talbot and Foster 2001). *M. oryzae* can act as a root pathogen on rice, barley and wheat (Sesma and Osbourn 2004) and some strains use the necrotrophic stage to infect *A. thaliana* (Park et al. 2008). These findings have implications for disease control strategies, fungal biology and its use as a model system.

Our results revealed that introduction of *PbCYP3* in the *M. oryzae* cyclophilin Δ*Cyp1* strain led to restoration of fungal colonization in plant tissues similar to wild-type levels. Deletion of this gene had as a result significant reduction of *M. oryzae* virulence, as earlier reported by Viaud et al. (2002). Our results showed that PbCYP3 was not able to fully restore the disease severity of Δ*Cyp1* to wild-type levels indicating that these two proteins do not exactly share the same function. We have to mention here that the infection biology of *P. brassicae* is very different compared to *M. oryzae* (Schwelm et al. 2016). Zoospores of *P. brassicae* encyst when attaching to a host root hair in the soil. The entering of root hair cells has been described to occur with the aid of a specialized mechanical structure used to inject a protoplast of the pathogen into the host cell (Aist and Williams 1971). PbCYP3 could possibly be involved in these processes since CYP1 in *M. oryzae* function in penetration peg formation and generation of appressoria turgor in the initial infection stages (Viaud et al. 2002).

Many aspects remain to be elucidated in the biology of *P. brassicae* not least to provide meaning to the extensive number of unknown function among the annotated *P. brassicae* -specific genes (> 50%) in the genome (Schwelm et al. 2015). Using heterologous test systems such as *M. oryzae* exploited in this study could help to accomplish such a task.

## Electronic supplementary material

Below is the link to the electronic supplementary material.

Supplementary dataset 1 Alignment of amino acid sequences used in phylogenetic analysis. Alignments were conducted using the CLUSTALW algorithm implemented in MegAlign software (DNASTAR, Madison). (TXT 13 KB)
